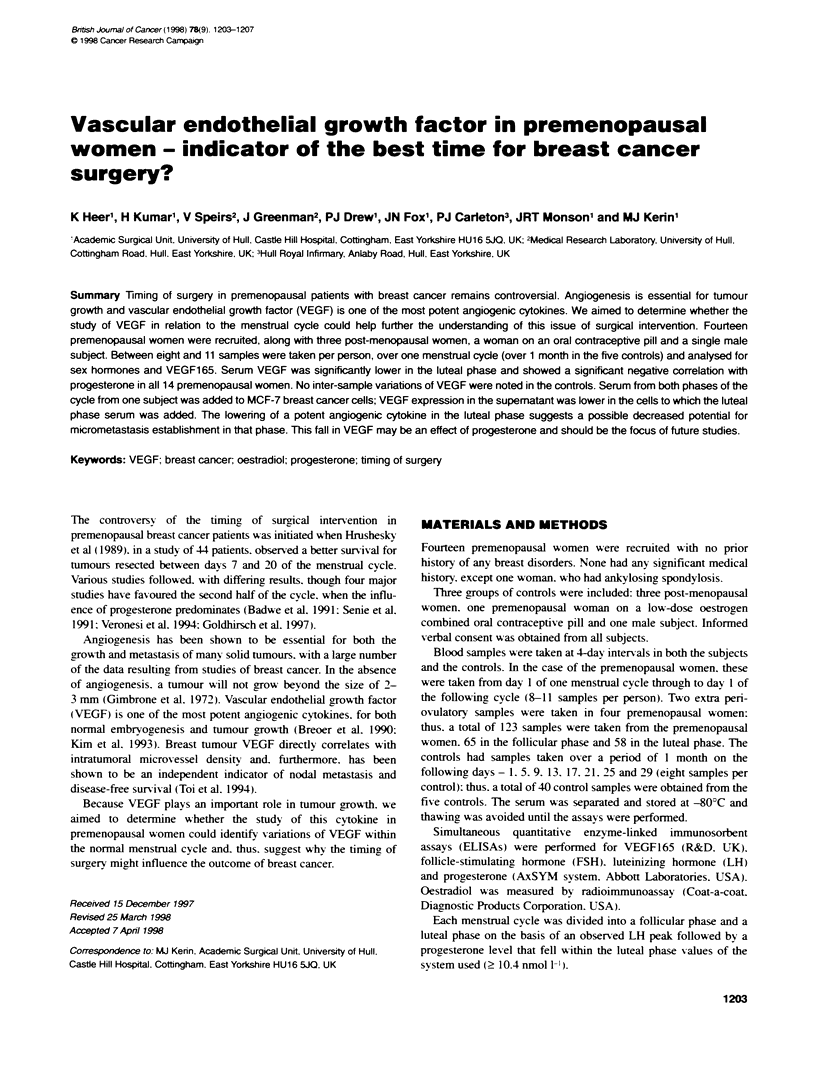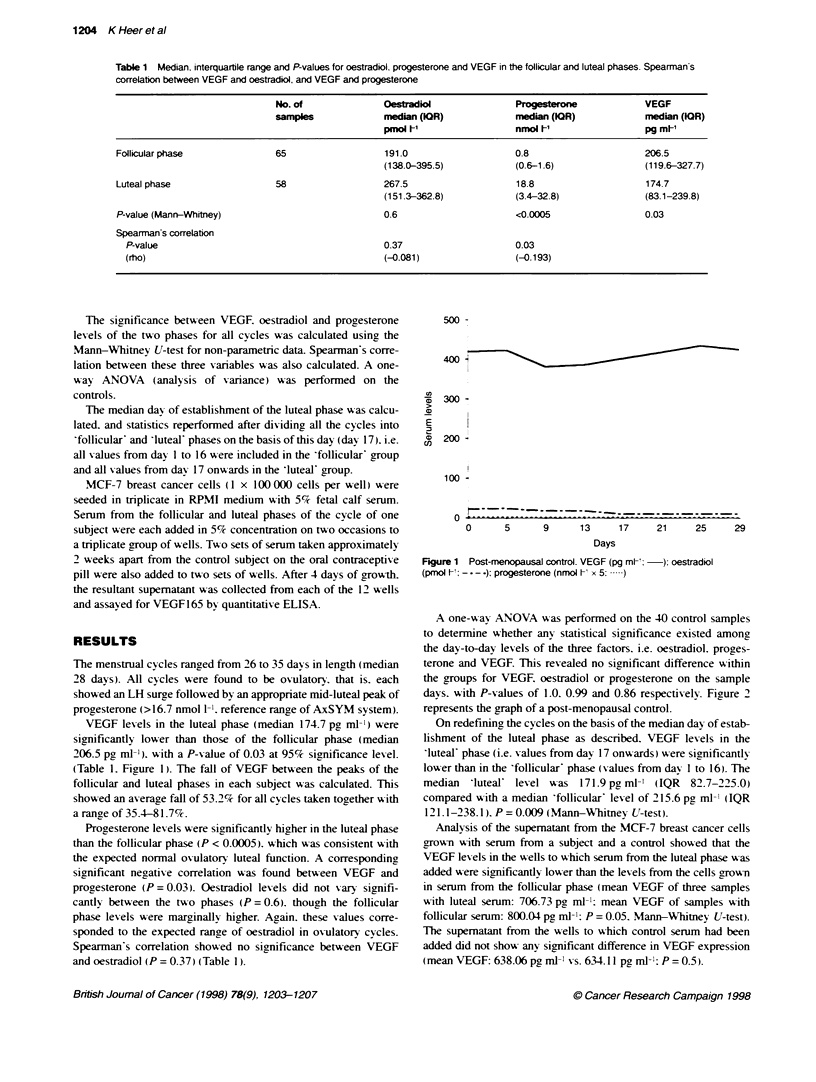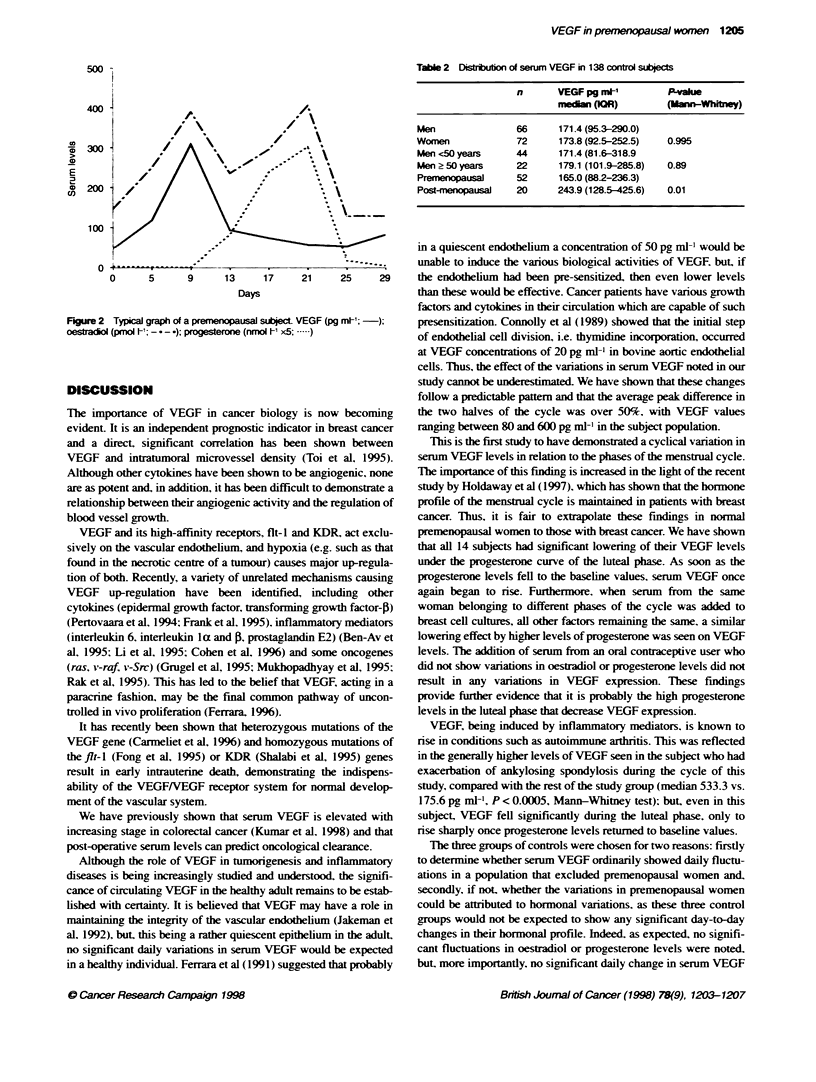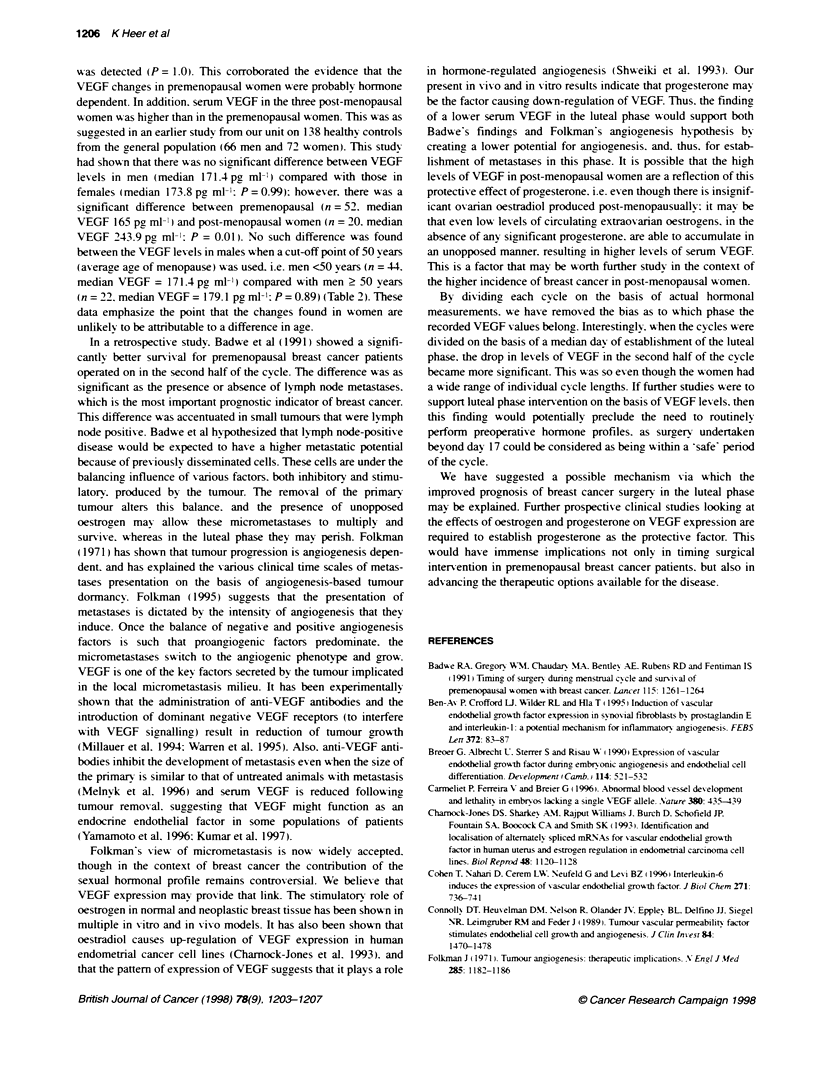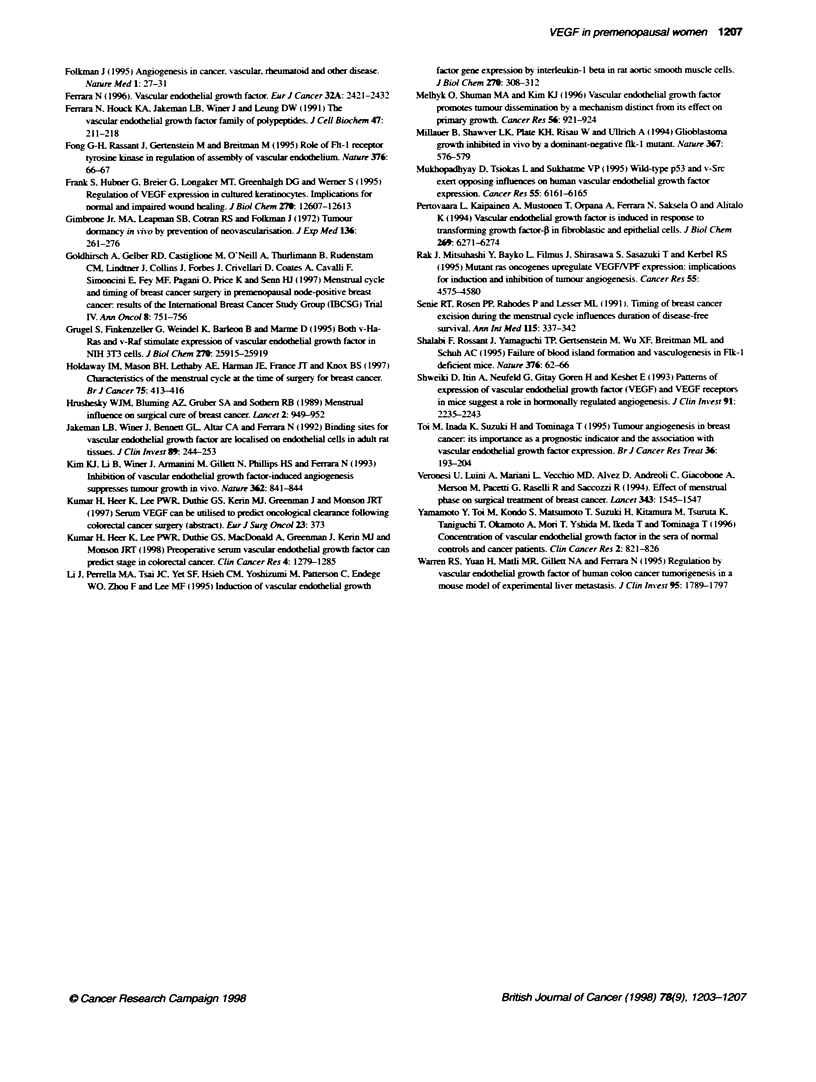# Vascular endothelial growth factor in premenopausal women--indicator of the best time for breast cancer surgery?

**DOI:** 10.1038/bjc.1998.655

**Published:** 1998-11

**Authors:** K. Heer, H. Kumar, V. Speirs, J. Greenman, P. J. Drew, J. N. Fox, P. J. Carleton, J. R. Monson, M. J. Kerin

**Affiliations:** Academic Surgical Unit, University of Hull, Castle Hill Hospital, Cottingham, East Yorkshire, UK.

## Abstract

Timing of surgery in premenopausal patients with breast cancer remains controversial. Angiogenesis is essential for tumour growth and vascular endothelial growth factor (VEGF) is one of the most potent angiogenic cytokines. We aimed to determine whether the study of VEGF in relation to the menstrual cycle could help further the understanding of this issue of surgical intervention. Fourteen premenopausal women were recruited, along with three post-menopausal women, a woman on an oral contraceptive pill and a single male subject. Between eight and 11 samples were taken per person, over one menstrual cycle (over 1 month in the five controls) and analysed for sex hormones and VEGF165. Serum VEGF was significantly lower in the luteal phase and showed a significant negative correlation with progesterone in all 14 premenopausal women. No inter-sample variations of VEGF were noted in the controls. Serum from both phases of the cycle from one subject was added to MCF-7 breast cancer cells; VEGF expression in the supernatant was lower in the cells to which the luteal phase serum was added. The lowering of a potent angiogenic cytokine in the luteal phase suggests a possible decreased potential for micrometastasis establishment in that phase. This fall in VEGF may be an effect of progesterone and should be the focus of future studies.


British Journal of Cancer (1998) 78(9). 1203–1207
© 1998 Cancer Research Campaign

### Vascular endothelial growth factor in premenopausal women – indicator of the best time for breast cancer surgery?

K Heer[1], H Kumar[1], V Speirs[2], J Greenman[2], PJ Drew[1], JN Fox[1], PJ Carleton[3], JRT Monson[1] and MJ Kerin[1]

[1]Academic Surgical Unit. University of Hull. Castle Hill Hospital. Cottingham. East Yorkshire HU16 5JQ. UK; [2]Medical Research Laboratory. University of Hull. Cottingham Road. Hull. East Yorkshire. UK; [3]Hull Royal Infirmary. Anlaby Road. Hull. East Yorkshire. UK

**Summary** Timing of surgery in premenopausal patients with breast cancer remains controversial. Angiogenesis is essential for tumour growth and vascular endothelial growth factor (VEGF) is one of the most potent angiogenic cytokines. We aimed to determine whether the study of VEGF in relation to the menstrual cycle could help further the understanding of this issue of surgical intervention. Fourteen premenopausal women were recruited, along with three post-menopausal women, a woman on an oral contraceptive pill and a single male subject. Between eight and 11 samples were taken per person, over one menstrual cycle (over 1 month in the five controls) and analysed for sex hormones and VEGF165. Serum VEGF was significantly lower in the luteal phase and showed a significant negative correlation with progesterone in all 14 premenopausal women. No inter-sample variations of VEGF were noted in the controls. Serum from both phases of the cycle from one subject was added to MCF-7 breast cancer cells; VEGF expression in the supernatant was lower in the cells to which the luteal phase serum was added. The lowering of a potent angiogenic cytokine in the luteal phase suggests a possible decreased potential for micrometastasis establishment in that phase. This fall in VEGF may be an effect of progesterone and should be the focus of future studies.

**Keywords:** VEGF; breast cancer; oestradiol; progesterone; timing of surgery

The controversy of the timing of surgical intervention in premenopausal breast cancer patients was initiated when Hrushesky et al (1989). in a study of 44 patients. observed a better survival for tumours resected between days 7 and 20 of the menstrual cycle. Various studies followed. with differing results. though four major studies have favoured the second half of the cycle. when the influence of progesterone predominates (Badwe et al. 1991; Senie et al. 1991; Veronesi et al. 1994; Goldhirsch et al. 1997).

Angiogenesis has been shown to be essential for both the growth and metastasis of many solid tumours. with a large number of the data resulting from studies of breast cancer. In the absence of angiogenesis. a tumour will not grow beyond the size of 2–3 mm (Gimbrone et al. 1972). Vascular endothelial growth factor (VEGF) is one of the most potent angiogenic cytokines. for both normal embryogenesis and tumour growth (Breoer et al. 1990; Kim et al. 1993). Breast tumour VEGF directly correlates with intratumoral microvessel density and. furthermore. has been shown to be an independent indicator of nodal metastasis and disease-free survival (Toi et al. 1994).

Because VEGF plays an important role in tumour growth. we aimed to determine whether the study of this cytokine in premenopausal women could identify variations of VEGF within the normal menstrual cycle and. thus. suggest why the timing of surgery might influence the outcome of breast cancer.

*Received 15 December 1997*
*Revised 25 March 1998*
*Accepted 7 April 1998*

*Correspondence to:* MJ Kerin. Academic Surgical Unit. University of Hull. Castle Hill Hospital. Cottingham. East Yorkshire HU16 5JQ. UK

### MATERIALS AND METHODS

Fourteen premenopausal women were recruited with no prior history of any breast disorders. None had any significant medical history. except one woman. who had ankylosing spondylosis.

Three groups of controls were included: three post-menopausal women. one premenopausal woman on a low-dose oestrogen combined oral contraceptive pill and one male subject. Informed verbal consent was obtained from all subjects.

Blood samples were taken at 4-day intervals in both the subjects and the controls. In the case of the premenopausal women. these were taken from day 1 of one menstrual cycle through to day 1 of the following cycle (8–11 samples per person). Two extra peri-ovulatory samples were taken in four premenopausal women: thus. a total of 123 samples were taken from the premenopausal women. 65 in the follicular phase and 58 in the luteal phase. The controls had samples taken over a period of 1 month on the following days – 1. 5. 9. 13. 17. 21. 25 and 29 (eight samples per control): thus. a total of 40 control samples were obtained from the five controls. The serum was separated and stored at –80°C and thawing was avoided until the assays were performed.

Simultaneous quantitative enzyme-linked immunosorbent assays (ELISAs) were performed for VEGF165 (R&D. UK). follicle-stimulating hormone (FSH). luteinizing hormone (LH) and progesterone (AxSYM system. Abbott Laboratories. USA). Oestradiol was measured by radioimmunoassay (Coat-a-coat. Diagnostic Products Corporation. USA).

Each menstrual cycle was divided into a follicular phase and a luteal phase on the basis of an observed LH peak followed by a progesterone level that fell within the luteal phase values of the system used ($\geq$ 10.4 nmol $l^{-1}$).

**Table 1** Median, interquartile range and *P*-values for oestradiol, progesterone and VEGF in the follicular and luteal phases. Spearman's correlation between VEGF and oestradiol, and VEGF and progesterone

| | No. of samples | Oestradiol median (IQR) pmol l$^{-1}$ | Progesterone median (IQR) nmol l$^{-1}$ | VEGF median (IQR) pg ml$^{-1}$ |
|---|---|---|---|---|
| Follicular phase | 65 | 191.0 (138.0–395.5) | 0.8 (0.6–1.6) | 206.5 (119.6–327.7) |
| Luteal phase | 58 | 267.5 (151.3–362.8) | 18.8 (3.4–32.8) | 174.7 (83.1–239.8) |
| *P*-value (Mann–Whitney) | | 0.6 | <0.0005 | 0.03 |
| Spearman's correlation | | | | |
| *P*-value | | 0.37 | 0.03 | |
| (rho) | | (–0.081) | (–0.193) | |

The significance between VEGF, oestradiol and progesterone levels of the two phases for all cycles was calculated using the Mann–Whitney *U*-test for non-parametric data. Spearman's correlation between these three variables was also calculated. A one-way ANOVA (analysis of variance) was performed on the controls.

The median day of establishment of the luteal phase was calculated, and statistics reperformed after dividing all the cycles into 'follicular' and 'luteal' phases on the basis of this day (day 17), i.e. all values from day 1 to 16 were included in the 'follicular' group and all values from day 17 onwards in the 'luteal' group.

MCF-7 breast cancer cells (1 × 100 000 cells per well) were seeded in triplicate in RPMI medium with 5% fetal calf serum. Serum from the follicular and luteal phases of the cycle of one subject were each added in 5% concentration on two occasions to a triplicate group of wells. Two sets of serum taken approximately 2 weeks apart from the control subject on the oral contraceptive pill were also added to two sets of wells. After 4 days of growth, the resultant supernatant was collected from each of the 12 wells and assayed for VEGF165 by quantitative ELISA.

### RESULTS

The menstrual cycles ranged from 26 to 35 days in length (median 28 days). All cycles were found to be ovulatory, that is, each showed an LH surge followed by an appropriate mid-luteal peak of progesterone (>16.7 nmol l$^{-1}$, reference range of AxSYM system).

VEGF levels in the luteal phase (median 174.7 pg ml$^{-1}$) were significantly lower than those of the follicular phase (median 206.5 pg ml$^{-1}$), with a *P*-value of 0.03 at 95% significance level. (Table 1, Figure 1). The fall of VEGF between the peaks of the follicular and luteal phases in each subject was calculated. This showed an average fall of 53.2% for all cycles taken together with a range of 35.4–81.7%.

Progesterone levels were significantly higher in the luteal phase than the follicular phase ($P < 0.0005$), which was consistent with the expected normal ovulatory luteal function. A corresponding significant negative correlation was found between VEGF and progesterone ($P = 0.03$). Oestradiol levels did not vary significantly between the two phases ($P = 0.6$), though the follicular phase levels were marginally higher. Again, these values corresponded to the expected range of oestradiol in ovulatory cycles. Spearman's correlation showed no significance between VEGF and oestradiol ($P = 0.37$) (Table 1).

**Figure 1** Post-menopausal control. VEGF (pg ml$^{-1}$; ——); oestradiol (pmol l$^{-1}$; – • – •); progesterone (nmol l$^{-1}$ × 5; ·····)

A one-way ANOVA was performed on the 40 control samples to determine whether any statistical significance existed among the day-to-day levels of the three factors, i.e. oestradiol, progesterone and VEGF. This revealed no significant difference within the groups for VEGF, oestradiol or progesterone on the sample days, with *P*-values of 1.0, 0.99 and 0.86 respectively. Figure 2 represents the graph of a post-menopausal control.

On redefining the cycles on the basis of the median day of establishment of the luteal phase as described, VEGF levels in the 'luteal' phase (i.e. values from day 17 onwards) were significantly lower than in the 'follicular' phase (values from day 1 to 16). The median 'luteal' level was 171.9 pg ml$^{-1}$ (IQR 82.7–225.0) compared with a median 'follicular' level of 215.6 pg ml$^{-1}$ (IQR 121.1–238.1), $P = 0.009$ (Mann–Whitney *U*-test).

Analysis of the supernatant from the MCF-7 breast cancer cells grown with serum from a subject and a control showed that the VEGF levels in the wells to which serum from the luteal phase was added were significantly lower than the levels from the cells grown in serum from the follicular phase (mean VEGF of three samples with luteal serum: 706.73 pg ml$^{-1}$; mean VEGF of samples with follicular serum: 800.04 pg ml$^{-1}$; $P = 0.05$, Mann–Whitney *U*-test). The supernatant from the wells to which control serum had been added did not show any significant difference in VEGF expression (mean VEGF: 638.06 pg ml$^{-1}$ vs. 634.11 pg ml$^{-1}$; $P = 0.5$).

© *Cancer Research Campaign 1998*

**Figure 2** Typical graph of a premenopausal subject. VEGF (pg ml⁻¹; ——); oestradiol (pmol l⁻¹; – • – •); progesterone (nmol l⁻¹ ×5; ·····)

### DISCUSSION

The importance of VEGF in cancer biology is now becoming evident. It is an independent prognostic indicator in breast cancer and a direct, significant correlation has been shown between VEGF and intratumoral microvessel density (Toi et al, 1995). Although other cytokines have been shown to be angiogenic, none are as potent and, in addition, it has been difficult to demonstrate a relationship between their angiogenic activity and the regulation of blood vessel growth.

VEGF and its high-affinity receptors, flt-1 and KDR, act exclusively on the vascular endothelium, and hypoxia (e.g. such as that found in the necrotic centre of a tumour) causes major up-regulation of both. Recently, a variety of unrelated mechanisms causing VEGF up-regulation have been identified, including other cytokines (epidermal growth factor, transforming growth factor-$\beta$) (Pertovaara et al, 1994; Frank et al, 1995), inflammatory mediators (interleukin 6, interleukin 1$\alpha$ and $\beta$, prostaglandin E2) (Ben-Av et al, 1995; Li et al, 1995; Cohen et al, 1996) and some oncogenes (*ras*, *v-raf*, *v-Src*) (Grugel et al, 1995; Mukhopadhyay et al, 1995; Rak et al, 1995). This has led to the belief that VEGF, acting in a paracrine fashion, may be the final common pathway of uncontrolled in vivo proliferation (Ferrara, 1996).

It has recently been shown that heterozygous mutations of the VEGF gene (Carmeliet et al, 1996) and homozygous mutations of the *flt*-1 (Fong et al, 1995) or KDR (Shalabi et al, 1995) genes result in early intrauterine death, demonstrating the indispensability of the VEGF/VEGF receptor system for normal development of the vascular system.

We have previously shown that serum VEGF is elevated with increasing stage in colorectal cancer (Kumar et al, 1998) and that post-operative serum levels can predict oncological clearance.

Although the role of VEGF in tumorigenesis and inflammatory diseases is being increasingly studied and understood, the significance of circulating VEGF in the healthy adult remains to be established with certainty. It is believed that VEGF may have a role in maintaining the integrity of the vascular endothelium (Jakeman et al, 1992), but, this being a rather quiescent epithelium in the adult, no significant daily variations in serum VEGF would be expected in a healthy individual. Ferrara et al (1991) suggested that probably in a quiescent endothelium a concentration of 50 pg ml⁻¹ would be unable to induce the various biological activities of VEGF, but, if the endothelium had been pre-sensitized, then even lower levels than these would be effective. Cancer patients have various growth factors and cytokines in their circulation which are capable of such presensitization. Connolly et al (1989) showed that the initial step of endothelial cell division, i.e. thymidine incorporation, occurred at VEGF concentrations of 20 pg ml⁻¹ in bovine aortic endothelial cells. Thus, the effect of the variations in serum VEGF noted in our study cannot be underestimated. We have shown that these changes follow a predictable pattern and that the average peak difference in the two halves of the cycle was over 50%, with VEGF values ranging between 80 and 600 pg ml⁻¹ in the subject population.

This is the first study to have demonstrated a cyclical variation in serum VEGF levels in relation to the phases of the menstrual cycle. The importance of this finding is increased in the light of the recent study by Holdaway et al (1997), which has shown that the hormone profile of the menstrual cycle is maintained in patients with breast cancer. Thus, it is fair to extrapolate these findings in normal premenopausal women to those with breast cancer. We have shown that all 14 subjects had significant lowering of their VEGF levels under the progesterone curve of the luteal phase. As soon as the progesterone levels fell to the baseline values, serum VEGF once again began to rise. Furthermore, when serum from the same woman belonging to different phases of the cycle was added to breast cell cultures, all other factors remaining the same, a similar lowering effect by higher levels of progesterone was seen on VEGF levels. The addition of serum from an oral contraceptive user who did not show variations in oestradiol or progesterone levels did not result in any variations in VEGF expression. These findings provide further evidence that it is probably the high progesterone levels in the luteal phase that decrease VEGF expression.

VEGF, being induced by inflammatory mediators, is known to rise in conditions such as autoimmune arthritis. This was reflected in the generally higher levels of VEGF seen in the subject who had exacerbation of ankylosing spondylosis during the cycle of this study, compared with the rest of the study group (median 533.3 vs. 175.6 pg ml⁻¹, $P < 0.0005$, Mann–Whitney test); but, even in this subject, VEGF fell significantly during the luteal phase, only to rise sharply once progesterone levels returned to baseline values.

The three groups of controls were chosen for two reasons: firstly to determine whether serum VEGF ordinarily showed daily fluctuations in a population that excluded premenopausal women and, secondly, if not, whether the variations in premenopausal women could be attributed to hormonal variations, as these three control groups would not be expected to show any significant day-to-day changes in their hormonal profile. Indeed, as expected, no significant fluctuations in oestradiol or progesterone levels were noted, but, more importantly, no significant daily change in serum VEGF

**Table 2** Distribution of serum VEGF in 138 control subjects

| | *n* | VEGF pg ml⁻¹ median (IQR) | *P*-value (Mann–Whitney) |
|---|---|---|---|
| Men | 66 | 171.4 (95.3–290.0) | |
| Women | 72 | 173.8 (92.5–252.5) | 0.995 |
| Men <50 years | 44 | 171.4 (81.6–318.9 | |
| Men ≥ 50 years | 22 | 179.1 (101.9–285.8) | 0.89 |
| Premenopausal | 52 | 165.0 (88.2–236.3) | |
| Post-menopausal | 20 | 243.9 (128.5–425.6) | 0.01 |

© Cancer Research Campaign 1998

was detected ($P = 1.0$). This corroborated the evidence that the VEGF changes in premenopausal women were probably hormone dependent. In addition, serum VEGF in the three post-menopausal women was higher than in the premenopausal women. This was as suggested in an earlier study from our unit on 138 healthy controls from the general population (66 men and 72 women). This study had shown that there was no significant difference between VEGF levels in men (median 171.4 pg ml$^{-1}$) compared with those in females (median 173.8 pg ml$^{-1}$; $P = 0.99$); however, there was a significant difference between premenopausal ($n = 52$, median VEGF 165 pg ml$^{-1}$) and post-menopausal women ($n = 20$, median VEGF 243.9 pg ml$^{-1}$; $P = 0.01$). No such difference was found between the VEGF levels in males when a cut-off point of 50 years (average age of menopause) was used, i.e. men <50 years ($n = 44$, median VEGF = 171.4 pg ml$^{-1}$) compared with men ≥ 50 years ($n = 22$, median VEGF = 179.1 pg ml$^{-1}$; $P = 0.89$) (Table 2). These data emphasize the point that the changes found in women are unlikely to be attributable to a difference in age.

In a retrospective study, Badwe et al (1991) showed a significantly better survival for premenopausal breast cancer patients operated on in the second half of the cycle. The difference was as significant as the presence or absence of lymph node metastases, which is the most important prognostic indicator of breast cancer. This difference was accentuated in small tumours that were lymph node positive. Badwe et al hypothesized that lymph node-positive disease would be expected to have a higher metastatic potential because of previously disseminated cells. These cells are under the balancing influence of various factors, both inhibitory and stimulatory, produced by the tumour. The removal of the primary tumour alters this balance, and the presence of unopposed oestrogen may allow these micrometastases to multiply and survive, whereas in the luteal phase they may perish. Folkman (1971) has shown that tumour progression is angiogenesis dependent, and has explained the various clinical time scales of metastases presentation on the basis of angiogenesis-based tumour dormancy. Folkman (1995) suggests that the presentation of metastases is dictated by the intensity of angiogenesis that they induce. Once the balance of negative and positive angiogenesis factors is such that proangiogenic factors predominate, the micrometastases switch to the angiogenic phenotype and grow. VEGF is one of the key factors secreted by the tumour implicated in the local micrometastasis milieu. It has been experimentally shown that the administration of anti-VEGF antibodies and the introduction of dominant negative VEGF receptors (to interfere with VEGF signalling) result in reduction of tumour growth (Millauer et al, 1994; Warren et al, 1995). Also, anti-VEGF antibodies inhibit the development of metastasis even when the size of the primary is similar to that of untreated animals with metastasis (Melnyk et al, 1996) and serum VEGF is reduced following tumour removal, suggesting that VEGF might function as an endocrine endothelial factor in some populations of patients (Yamamoto et al, 1996; Kumar et al, 1997).

Folkman's view of micrometastasis is now widely accepted, though in the context of breast cancer the contribution of the sexual hormonal profile remains controversial. We believe that VEGF expression may provide that link. The stimulatory role of oestrogen in normal and neoplastic breast tissue has been shown in multiple in vitro and in vivo models. It has also been shown that oestradiol causes up-regulation of VEGF expression in human endometrial cancer cell lines (Charnock-Jones et al, 1993), and that the pattern of expression of VEGF suggests that it plays a role in hormone-regulated angiogenesis (Shweiki et al, 1993). Our present in vivo and in vitro results indicate that progesterone may be the factor causing down-regulation of VEGF. Thus, the finding of a lower serum VEGF in the luteal phase would support both Badwe's findings and Folkman's angiogenesis hypothesis by creating a lower potential for angiogenesis, and, thus, for establishment of metastases in this phase. It is possible that the high levels of VEGF in post-menopausal women are a reflection of this protective effect of progesterone, i.e. even though there is insignificant ovarian oestradiol produced post-menopausually; it may be that even low levels of circulating extraovarian oestrogens, in the absence of any significant progesterone, are able to accumulate in an unopposed manner, resulting in higher levels of serum VEGF. This is a factor that may be worth further study in the context of the higher incidence of breast cancer in post-menopausal women.

By dividing each cycle on the basis of actual hormonal measurements, we have removed the bias as to which phase the recorded VEGF values belong. Interestingly, when the cycles were divided on the basis of a median day of establishment of the luteal phase, the drop in levels of VEGF in the second half of the cycle became more significant. This was so even though the women had a wide range of individual cycle lengths. If further studies were to support luteal phase intervention on the basis of VEGF levels, then this finding would potentially preclude the need to routinely perform preoperative hormone profiles, as surgery undertaken beyond day 17 could be considered as being within a 'safe' period of the cycle.

We have suggested a possible mechanism via which the improved prognosis of breast cancer surgery in the luteal phase may be explained. Further prospective clinical studies looking at the effects of oestrogen and progesterone on VEGF expression are required to establish progesterone as the protective factor. This would have immense implications not only in timing surgical intervention in premenopausal breast cancer patients, but also in advancing the therapeutic options available for the disease.

### REFERENCES

Badwe RA, Gregory WM, Chaudary MA, Bentley AE, Rubens RD and Fentiman IS (1991) Timing of surgery during menstrual cycle and survival of premenopausal women with breast cancer. *Lancet* 115: 1261–1264

Ben-Av P, Crofford LJ, Wilder RL and Hla T (1995) Induction of vascular endothelial growth factor expression in synovial fibroblasts by prostaglandin E and interleukin-1: a potential mechanism for inflammatory angiogenesis. *FEBS Lett* **372**: 83–87

Breier G, Albrecht U, Sterrer S and Risau W (1990) Expression of vascular endothelial growth factor during embryonic angiogenesis and endothelial cell differentiation. *Development (Camb.)* **114**: 521–532

Carmeliet P, Ferreira V and Breier G (1996). Abnormal blood vessel development and lethality in embryos lacking a single VEGF allele. *Nature* **380**: 435–439

Charnock-Jones DS, Sharkey AM, Rajput Williams J, Burch D, Schofield JP, Fountain SA, Boocock CA and Smith SK (1993). Identification and localisation of alternately spliced mRNAs for vascular endothelial growth factor in human uterus and estrogen regulation in endometrial carcinoma cell lines. *Biol Reprod* **48**: 1120–1128

Cohen T, Nahari D, Cerem LW, Neufeld G and Levi BZ (1996) Interleukin-6 induces the expression of vascular endothelial growth factor. *J Biol Chem* **271**: 736–741

Connolly DT, Heuvelman DM, Nelson R, Olander JV, Eppley BL, Delfino JJ, Siegel NR, Leimgruber RM and Feder J (1989). Tumour vascular permeability factor stimulates endothelial cell growth and angiogenesis. *J Clin Invest* **84**: 1470–1478

Folkman J (1971). Tumour angiogenesis: therapeutic implications. *N Engl J Med* **285**: 1182–1186

© Cancer Research Campaign 1998

Folkman J (1995) Angiogenesis in cancer, vascular, rheumatoid and other disease. *Nature Med* **1**: 27–31

Ferrara N (1996). Vascular endothelial growth factor. *Eur J Cancer* **32A**: 2421–2432

Ferrara N, Houck KA, Jakeman LB, Winer J and Leung DW (1991) The vascular endothelial growth factor family of polypeptides. *J Cell Biochem* **47**: 211–218

Fong G-H, Rassant J, Gertenstein M and Breitman M (1995) Role of Flt-1 receptor tyrosine kinase in regulation of assembly of vascular endothelium. *Nature* **376**: 66–67

Frank S, Hubner G, Breier G, Longaker MT, Greenhalgh DG and Werner S (1995) Regulation of VEGF expression in cultured keratinocytes. Implications for normal and impaired wound healing. *J Biol Chem* **270**: 12607–12613

Gimbrone Jr, MA, Leapman SB, Cotran RS and Folkman J (1972) Tumour dormancy *in vivo* by prevention of neovascularisation. *J Exp Med* **136**: 261–276

Goldhirsch A, Gelber RD, Castiglione M, O'Neill A, Thurlimann B, Rudenstam CM, Lindtner J, Collins J, Forbes J, Crivellari D, Coates A, Cavalli F, Simoncini E, Fey MF, Pagani O, Price K and Senn HJ (1997) Menstrual cycle and timing of breast cancer surgery in premenopausal node-positive breast cancer: results of the International Breast Cancer Study Group (IBCSG) Trial IV. *Ann Oncol* **8**: 751–756

Grugel S, Finkenzeller G, Weindel K, Barleon B and Marme D (1995) Both v-Ha-Ras and v-Raf stimulate expression of vascular endothelial growth factor in NIH 3T3 cells. *J Biol Chem* **270**: 25915–25919

Holdaway IM, Mason BH, Lethaby AE, Harman JE, France JT and Knox BS (1997) Characteristics of the menstrual cycle at the time of surgery for breast cancer. *Br J Cancer* **75**: 413–416

Hrushesky WJM, Bluming AZ, Gruber SA and Sothern RB (1989) Menstrual influence on surgical cure of breast cancer. *Lancet* **2**: 949–952

Jakeman LB, Winer J, Bennett GL, Altar CA and Ferrara N (1992) Binding sites for vascular endothelial growth factor are localised on endothelial cells in adult rat tissues. *J Clin Invest* **89**: 244–253

Kim KJ, Li B, Winer J, Armanini M, Gillett N, Phillips HS and Ferrara N (1993) Inhibition of vascular endothelial growth factor-induced angiogenesis suppresses tumour growth in vivo. *Nature* **362**: 841–844

Kumar H, Heer K, Lee PWR, Duthie GS, Kerin MJ, Greenman J and Monson JRT (1997) Serum VEGF can be utilised to predict oncological clearance following colorectal cancer surgery (abstract). *Eur J Surg Oncol* **23**: 373

Kumar H, Heer K, Lee PWR, Duthie GS, MacDonald A, Greenman J, Kerin MJ and Monson JRT (1998) Preoperative serum vascular endothelial growth factor can predict stage in colorectal cancer. *Clin Cancer Res* **4**: 1279–1285

Li J, Perrella MA, Tsai JC, Yet SF, Hsieh CM, Yoshizumi M, Patterson C, Endege WO, Zhou F and Lee MF (1995) Induction of vascular endothelial growth factor gene expression by interleukin-1 beta in rat aortic smooth muscle cells. *J Biol Chem* **270**: 308–312

Melhyk O, Shuman MA and Kim KJ (1996) Vascular endothelial growth factor promotes tumour dissemination by a mechanism distinct from its effect on primary growth. *Cancer Res* **56**: 921–924

Millauer B, Shawver LK, Plate KH, Risau W and Ullrich A (1994) Glioblastoma growth inhibited in vivo by a dominant-negative flk-1 mutant. *Nature* **367**: 576–579

Mukhopadhyay D, Tsiokas L and Sukhatme VP (1995) Wild-type p53 and v-Src exert opposing influences on human vascular endothelial growth factor expression. *Cancer Res* **55**: 6161–6165

Pertovaara L, Kaipainen A, Mustonen T, Orpana A, Ferrara N, Saksela O and Alitalo K (1994) Vascular endothelial growth factor is induced in response to transforming growth factor-β in fibroblastic and epithelial cells. *J Biol Chem* **269**: 6271–6274

Rak J, Mitsuhashi Y, Bayko L, Filmus J, Shirasawa S, Sasazuki T and Kerbel RS (1995) Mutant ras oncogenes upregulate VEGF/VPF expression: implications for induction and inhibition of tumour angiogenesis. *Cancer Res* **55**: 4575–4580

Senie RT, Rosen PP, Rahodes P and Lesser ML (1991). Timing of breast cancer excision during the menstrual cycle influences duration of disease-free survival. *Ann Int Med* **115**: 337–342

Shalabi F, Rossant J, Yamaguchi TP, Gertsenstein M, Wu XF, Breitman ML and Schuh AC (1995) Failure of blood island formation and vasculogenesis in Flk-1 deficient mice. *Nature* **376**: 62–66

Shweiki D, Itin A, Neufeld G, Gitay Goren H and Keshet E (1993) Patterns of expression of vascular endothelial growth factor (VEGF) and VEGF receptors in mice suggest a role in hormonally regulated angiogenesis. *J Clin Invest* **91**: 2235–2243

Toi M, Inada K, Suzuki H and Tominaga T (1995) Tumour angiogenesis in breast cancer: its importance as a prognostic indicator and the association with vascular endothelial growth factor expression. *Br J Cancer Res Treat* **36**: 193–204

Veronesi U, Luini A, Mariani L, Vecchio MD, Alvez D, Andreoli C, Giacobone A, Merson M, Pacetti G, Raselli R and Saccozzi R (1994). Effect of menstrual phase on surgical treatment of breast cancer. *Lancet* **343**: 1545–1547

Yamamoto Y, Toi M, Kondo S, Matsumoto T, Suzuki H, Kitamura M, Tsuruta K, Taniguchi T, Okamoto A, Mori T, Yshida M, Ikeda T and Tominaga T (1996) Concentration of vascular endothelial growth factor in the sera of normal controls and cancer patients. *Clin Cancer Res* **2**: 821–826

Warren RS, Yuan H, Matli MR, Gillett NA and Ferrara N (1995) Regulation by vascular endothelial growth factor of human colon cancer tumorigenesis in a mouse model of experimental liver metastasis. *J Clin Invest* **95**: 1789–1797

© Cancer Research Campaign 1998